# Total saponins of *Bolbostemma paniculatum* (maxim.) Franquet exert antitumor activity against MDA-MB-231 human breast cancer cells via inhibiting PI3K/Akt/mTOR pathway

**DOI:** 10.1186/s12906-019-2708-0

**Published:** 2019-11-08

**Authors:** Jian-Wei Dou, Rong-Guo Shang, Xiao-Qin Lei, Kang-Le Li, Zhan-Zi Guo, Kai Ye, Xiao-Juan Yang, Yu-Wei Li, Yun-Yun Zhou, Jia Yao, Qian Huang

**Affiliations:** 1 School of Pharmacy, Xi’an Jiaotong University, Xi’an, Shaanxi 710061 People’s Republic of China; 2 Shaanxi Key Laboratory of “Qiyao” Resources And Anti-tumor Activities, Xi’an, Shaanxi 710061 People’s Republic of China; 3 Department of Ophthalmology, Affiliated Guangren Hospital of Xi’an Jiaotong University, Xi’an, Shaanxi 710004 People’s Republic of China; 4 Department of Ophthalmology, Xi’an No.4 Hospital, Xi’an, Shaanxi 710004 People’s Republic of China; 5 School of Basic Medicine, Gansu University of Chinese Medicine, Lanzhou, Gansu 730000 People’s Republic of China; 6 Xi’an Hospital of Traditional Chinese Medicine Affiliated to Shaanxi University of Chinese Medicine, Xi’an, Shaanxi 710021 People’s Republic of China

**Keywords:** *Bolbostemma paniculatum* (maxim.) Franquet, Total saponins, MDA-MB-231 cells, PI3K/Akt/mTOR, signaling pathway

## Abstract

**Background:**

The aim of the present study was to examine the effects of the *Bolbostemma paniculatum* (Maxim.) Franquet (BP) active compound, BP total saponins (BPTS), on MDA-MB-231 cells, and investigate the underlying mechanism regarding BPTS-mediated attenuation of the PI3K/Akt/mTOR pathway.

**Methods:**

The effect of BPTS on cytotoxicity, induction of apoptosis and migration on MDA-MB-231 cells at three different concentrations was investigated. A CCK-8 assay, wound-healing assay and flow cytometry were used to demonstrate the effects of BPTS. Additionally, expression of the primary members of the PI3K/Akt/mTOR signaling pathway was assessed using western blotting. To verify the underlying mechanisms, a PI3K inhibitor and an mTOR inhibitor were used.

**Results:**

BPTS inhibited proliferation of MDA-MB-231 cells with an IC_50_ value of 10 μg/mL at 48 h. BPTS inhibited migration of MDA-MB-231 cells, and the western blot results demonstrated that BPTS reduced p-PI3K, p-Akt and p-mTOR protein expression levels in MDA-MB-231 cells. Additionally, the results were confirmed using a PI3K inhibitor and an mTOR inhibitor. BPTS decreased proliferation and migration of MDA-MB-231 cells possibly through inhibiting the PI3K/Akt/mTOR signaling pathway.

**Conclusions:**

The results highlight the therapeutic potential of BPTS for treating patients with triple-negative breast cancer.

## Background

Because of the high incidence rate and complexity of the disease, breast cancer is the second largest cause of cancer-associated deaths in women worldwide. Triple-negative breast cancer (TNBC) with characteristics of early invasion, a propensity to metastasize and a relatively high rate of mortality amongst all breast cancer subtypes, accounts for 15–20% of all breast cancer cases [1]. In total, four main subgroups of human breast tumors have been identified, luminal A (LA), luminal B (LB), human epidermal growth factor receptor 2 (Her2)-overexpressing and TNBC [2]. Patients with TNBC do not often benefit from currently available therapeutics due to the complexity and diversity of TNBC [3]. Treatment regimens currently used to treat patients with TNBC present with many issues, including poor prognosis, expense and severe pain [4, 5]. Therefore, the development of novel therapeutics with fewer side effects and a relatively lower cost of production is required.

Traditional Chinese medicine may be viable alternative as patients may exhibit fewer side effects and are typically more economical [6–8]. Additionally, traditional Chinese medicines have been demonstrated to prevent and treat a number of diseases and may possess antiviral, anti-inflammatory, anticancer and immunosuppressive properties [9–11]. *Bolbostemma paniculatum* (Maxim.) Franquet (BP), referred to as Tu-bei-mu in China, is a member of Cucurbitaceae family [12]. BP has been used to treat breast cancer for > 200 years following its inclusion in “*Waike Zhengzhi Quansheng Ji*” during the Qing dynasty [13]. Previous studies have demonstrated that BP has a number of constituents, including saponins, organic acids, sterols, and alkaloids, all of which possess beneficial pharmacological properties relevant to treating different types of cancer [14, 15]. Total saponin is the primary active compound in BP and has been reported to inhibit the proliferation of MDA-MB-231 cells [16, 17]. However, the mechanisms of total saponin action are not yet understood.

The PI3K/Akt/mTOR signaling pathway serves an important role in proliferation and metastasis of tumor cells, and angiogenesis [18]. In addition, the PI3K/Akt/mTOR signaling pathway has also been implicated in resistance to radiotherapy and chemotherapy [19]. Furthermore, the signaling pathway is frequently activated and serves a vital role in the progression of TNBC [20, 21]. Therefore, members of this pathway present as potential therapeutic targets for treating patients with TNBC. An increase in PI3K expression results in increased phosphorylation of Akt [22]. mTOR is a downstream target of p-Akt, which, when activated further regulates protein synthesis and promotes cell proliferation [23]. Therefore, the present study examined the effects of BP total saponin (BPTS) on TNBC and in particular the effects on the PI3K/Akt/mTOR signaling pathway.

The MDA-MB-231 cell line is the most common and representative cell line used to study TNBC [24]. It is a TNBC cell line and its use may contribute to a better understanding of molecular mechanisms of the initiation, proliferation and other aspects of TNBC. In the present study, MDA-MB-231 cells were used to examine the effects of BPTS on TNBC and the possible underlying mechanisms.

## Materials and methods

### Reagents

BP was obtained from Beijing Tongrentang Group Co., Ltd. (Xi’an, China; specimen no. 20170306), and the sample identity was confirmed according to Chinese Pharmacopoeia. Dimethyl sulfoxide (DMSO), LY294002 and rapamycin were purchased from Sigma-Aldrich; Merck KGaA (Darmstadt, Germany). Cell Counting Kit-8 (CCK-8) was obtained from 7Sea BioTech (Shanghai, China). Fetal bovine serum (FBS) and L-15 medium were purchased from Gibco; Thermo Fisher Scientific, Inc. (Waltham, MA, USA). All antibodies used for western blotting were purchased from CST Biological Reagents Co., Ltd. (Shanghai, China). Western blotting reagents were purchased from Hat BioTech Co., Ltd. (Xi’an, China). Apoptosis Detection kits were purchased from Nanjing KGI Biological Technology Development Co., Ltd. (Nanjing, China). Petroleum ether and n-butanol were purchased from Kemiou Chemical Reagent Co., Ltd. (Tianjin, China).

### Preparation of BPTS

**To** prepare BPTS, 95% ethanol was applied and refluxed with 1.0 kg dried BP for 2 h at 80 °C. Then, the ethanol was removed by rotary evaporator. The concentrated extract was resuspended in water. The water fraction was partitioned with petroleum ether followed by n-butanol. The n-butanol fraction was allowed to evaporate, and the resultant sample was vacuum dried and crushed into a powder. Total saponin content was determined by UV spectroscopy at 472 nm (Persee, TU-1810, Beijing, China). Using this protocol, 36 g of a yellowish powder was obtained (yield, 3.6%; total saponin content, 79.1%). The resultant powder was used for all subsequent experiments.

### Cell culture

MDA-MB-231 cells were purchased from OBiO Technology Corp., Ltd. (Shanghai, China), and grown in L-15 supplemented with 10% FBS and 1% penicillin-streptomycin in a saturated humidity incubator (37 °C, 5%CO_2_).

### CCK-8 assay

Cell viability was determined using a CCK-8 assay according to the manufacturer’s protocol. MDA-MB-231 cells were seeded at a density of 5 × 10^3^ cells/well in a 96-well culture plate for 24 h after which the medium was changed and BPTS (5, 10, 15 μg/mL) was added and cultured for a further for 24, 48 or 72 h. 20 μl CCK-8 reagent was added to each well and incubated for 4 h. The optical density at 450 nm was measured using a Variskan® Flash microplate reader (Thermo Scientific Fisher, Inc.).

### Apoptosis analysis by flow cytometry

Annexin V-fluoroscein isothiocyanate (FITC)/propidium iodide (PI) double labeling was used to determine the apoptosis-inducing effect of BPTS on MDA-MB-231 cells. MDA-MB-231 cells were seeded into 6-well plates at a density of 1 × 10^6^ cells per well, and incubated with BPTS for 48 h after which cells were trypsinized, collected and incubated with FITC-conjugated Annexin V and PI according to the manufacturer’s instructions (BD Biosciences, San Jose, CA, USA). Apoptosis was measured by flow cytometry (BD FACSCalibur) and the data were analyzed using Cell Quest Research version 6.0 (BD Biosciences).

### Western blot analysis

Proteins were extracted from MDA-MB-231 cells using RIPA buffer (Hart Biologicals, Ltd.) *and the concentration was measured* using a bicinchoninic acid assay. A total of 30 μg protein was loaded into each lane of a 10% polyacrylamide gel and separated by SDS-PAGE. After the proteins were resolved, they were transferred to a PVDF membrane (EMD Millipore, Billerica, MA, USA). Membranes were blocked with 5% non-fat dried milk, incubated with anti-PI3K (1∶1000), anti-AKT (1:1000), anti-mTOR (1:1000), anti-p-PI3K (1:1000), anti-p-AKT (1∶1000), anti-p-mTOR (1:1000) antibodies overnight at 4 °C, and then incubated with the horseradish peroxidase-conjugated secondary antibodies (1:10000) for 2 h at room temperature. Enhanced chemiluminescence detection kits (EMD Millipore) were used to visualize bands, and intensity of the bands were quantified by 1.8.0 version ImageJ (National Institutes of Health, Bethesda, MD, USA). Besides, actin was used to quantify the amount and integrity of the proteins. When inhibitors were employed, cells were pretreated for 3 h with inhibitor (LY294002, 20 μM; Rapamycin, 20 μM) before the addition of BPTS.

### Wound healing assay

Wound healing assays were performed to determine the effects of BPTS on migration. A total of 5 × 10^4^ cells were plated in each well of a 6-well plate. Once the confluence had reach > 90% a 200 μl pipette tip was used to scratch five wounds in the cell layer. PBS was used to gently remove floating cells, and serum-free medium containing the aforementioned concentrations of BPTS was added to each well. The wounds were imaged at 0, 12, 24 and 48 h after scratching.

Migration rate (%) = (Scratch distance at 0 h - scratch distance at indicated time)/Scratch distance at 0 h × 100%.

### Transwell migration assay

A total of 3 × 10^4^ cells were plated with or without BPTS into the upper chamber of a 24-well Transwell chamber separated by a polycarbonate filter. Serum-free medium was added to the upper chamber and medium containing 10% FBS was added to the bottom chamber. After 48 h, the cells on the top side of the inserts were scraped off, and the Transwell filters were stained with 0.1% crystal violet for 0.5 h at room temperature and counted using an inverted microscope (Nikon, T*i*, Japan).

### Statistical analyses

Data are expressed as the mean ± standard error of *mean*. Statistical analyses were performed using one-way ANOVA in SPSS version 18.0 (*IBM Corporation, Armonk, NY, USA*) and Prism 5.0 (GraphPad Software, Inc., La Jolla, CA, USA). *P* < 0.05 was considered to indicate a statistically *significant.*

## Results

### Inhibitory effect of BPTS on proliferation of MDA-MB-231 cells

**Fig. 1:**
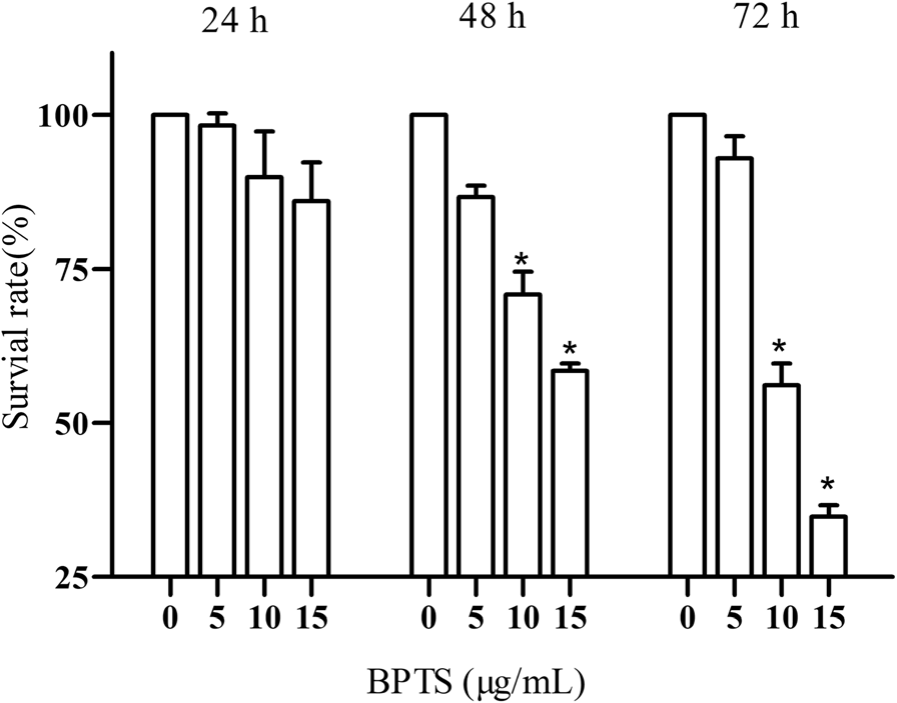
Effect of BPTS on proliferation of MDA-MB-231 cells. Results are presented as a percentage of the 0 μg/mL BPTS group. ^*^P < 0.05 vs. 0 μg/mL of BPTS

Cell viability was assessed to determine the effect of BPTS on proliferation. BPTS significantly inhibited the proliferation of MDA-MB-231 cells in a dose- and time-dependent manner (Fig. 1). The results show that cell viability was decreased following treatment with 10 and 15 μg/mL BPTS after 48 and 72 h. Therefore, for all subsequent experiments, cells were treated with 5, 10 and 15 μg/mL BPTS for 48 h.

### Apoptosis-inducing effect of BPTS on MDA-MB-231 cells

**Fig. 2:**
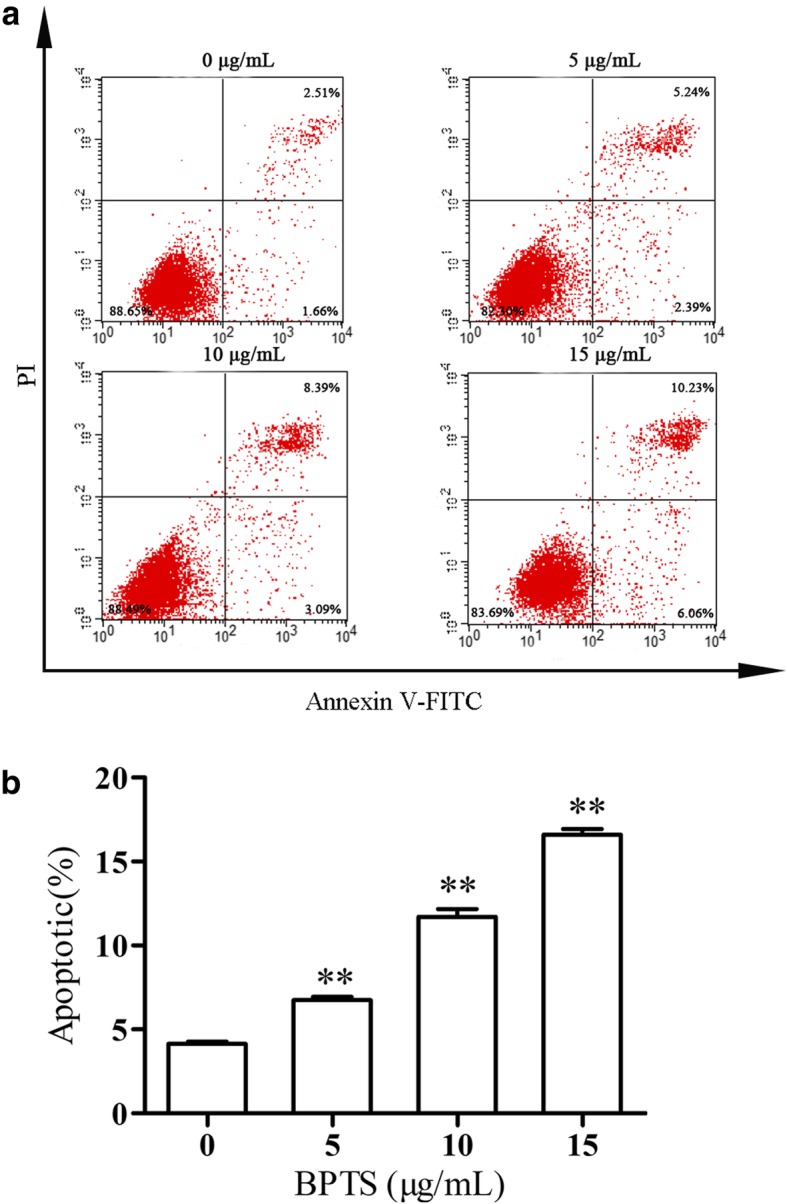
Flow cytometry analysis of apoptosis in MDA-MB-231 cells. **a** Representative images of flow cytometry. **b** Apoptotic rates in cells treated with the stated concentrations of BPTS. ^**^P < 0.01 vs. 0 μg/mL of BPTS

The proportion of apoptotic cells increased significantly concurrent with an increase in BPTS concentration (Fig. 2). The proportion of apoptotic cell with 5, 10 and 15 μg/mL BPTS was 7.54, 11.48 and 16.29%, respectively. These results suggest that BPTS induced apoptosis in MDA-MB-231 cells.

### Pathway-related mechanisms of the apoptotic effects of BPTS

**Fig. 3:**
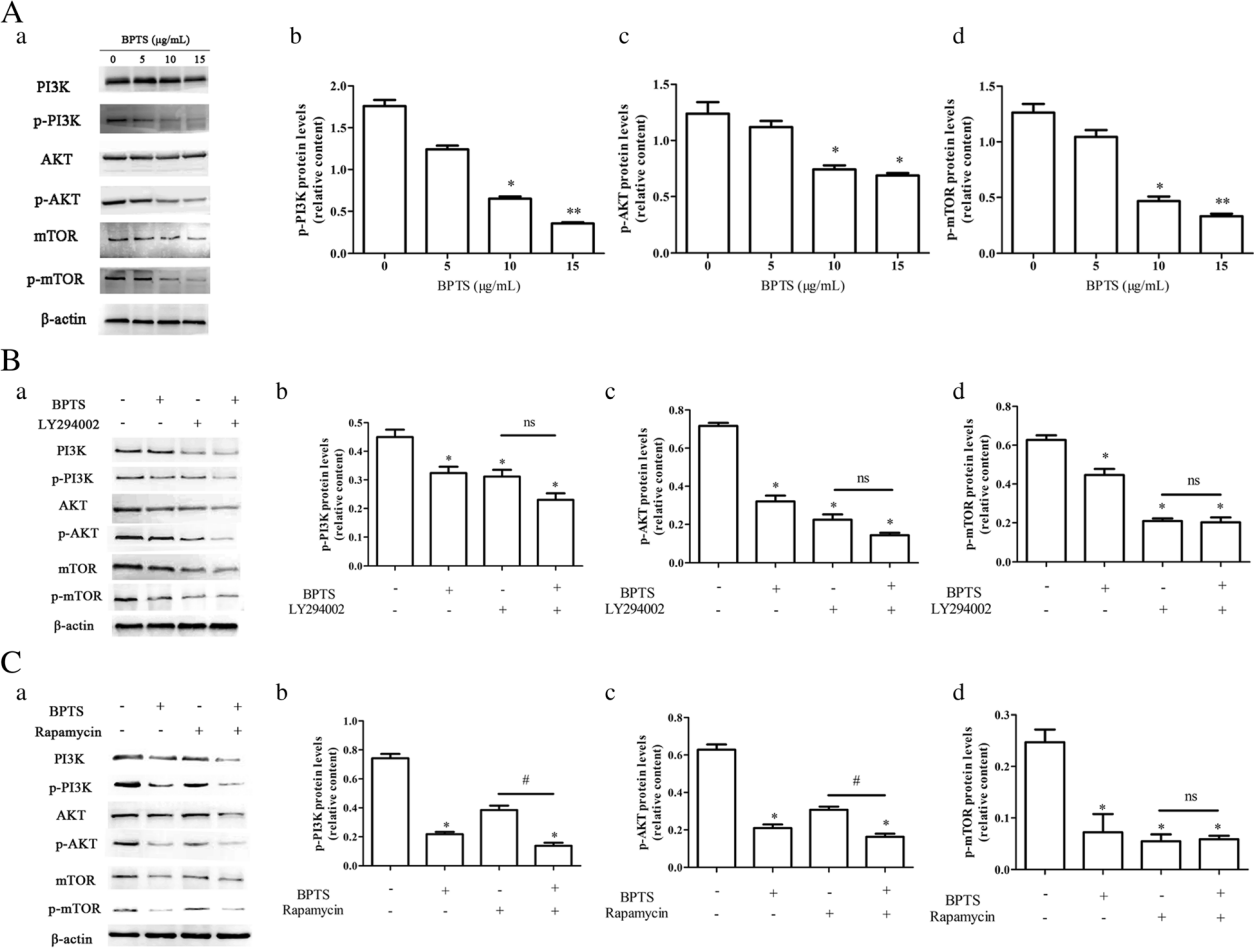
Protein expression levels of p-PI3K, p-Akt, p-mTOR in MDA-MB-231 cells treated with BPTS. (**A**) Western blot of the proteins in cells treated with BPTS alone, (**B**) BPTS + LY294002 and (**C**) BPTS + rapamycin. (**a**) Representative blot of expression. (**b**), (**c**) and (**d**) Quantification of expression normalized to expression of β-actin. ^*^P < 0.05 vs. 0 μg/mL of BPTS; ns, not significant

To investigate the role of PI3K/Akt/mTOR pathway in BPTS-induced apoptosis, the protein expression levels of PI3K, Akt, mTOR, p-PI3K, p-Akt and p-mTOR were examined by western blot. The levels of p-PI3K, p-Akt and p-mTOR in MDA-MB-231 cells treated with BPTS were decreased (Fig. 3). To confirm that BPTS induced apoptosis via the PI3K/Akt/mTOR pathway, cells were treated with a PI3K inhibitor (LY294002) prior to treatment with BPTS. Treatment with LY294002 reduced the levels of Akt phosphorylation. As Akt has many downstream targets, to determine whether mTOR was involved in BPTS-induced apoptosis, rapamycin, which is an mTOR inhibitor, was added to verify the mechanisms. The levels of p-mTOR was decreased when rapamycin intervening, the same as BPTS. These data suggest that BPTS initiates apoptosis by inhibiting the PI3K/Akt/mTOR signaling pathway.

### BPTS decreases the migratory capacity of MDA-MB-231 cells

**Fig. 4:**
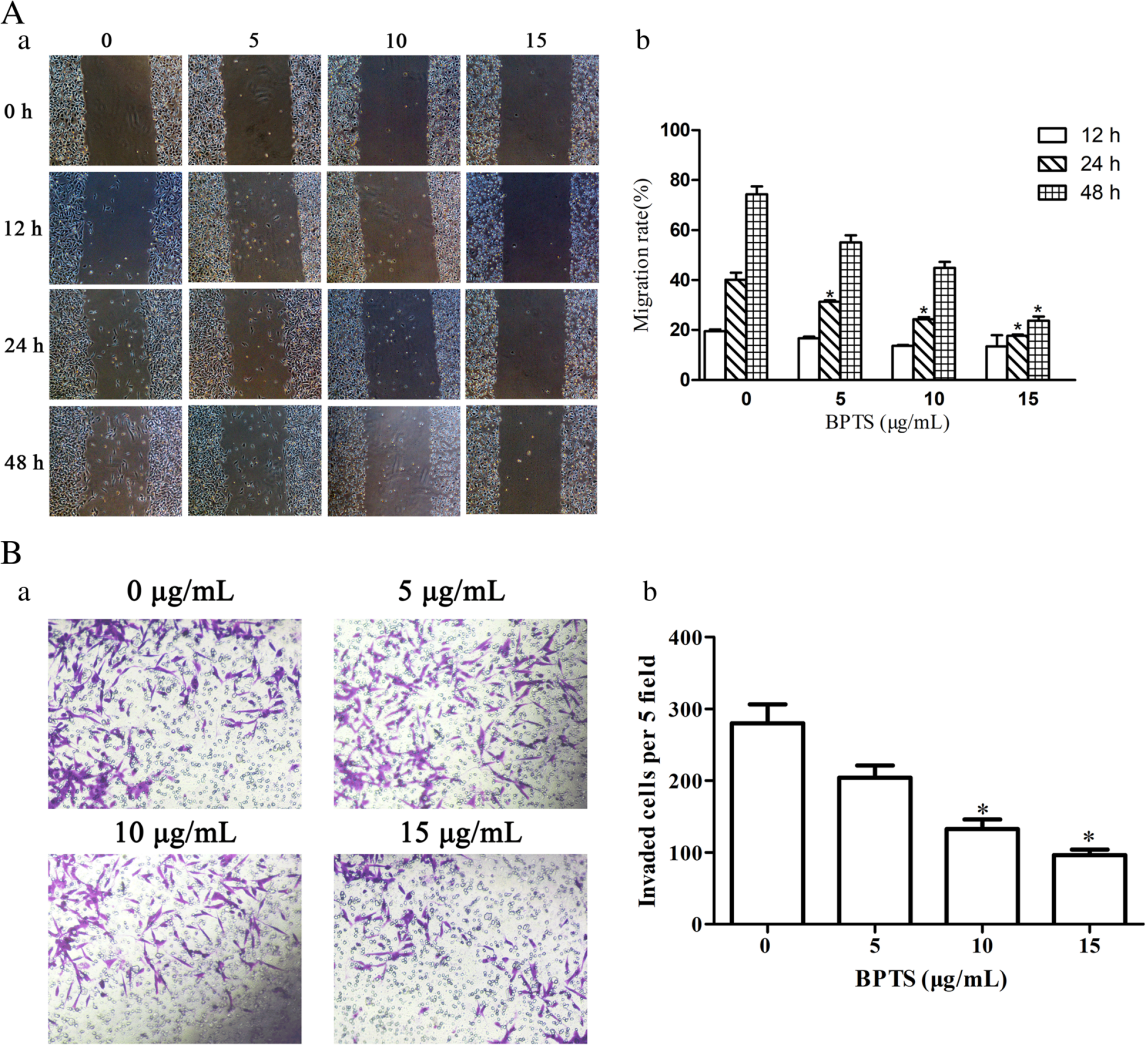
Representative images from wound healing and transwell migration assays of cells treated with BPTS were obtained after treatment with different concentrations of BPTS. (**a**) Representative image of the wound-healing and transwell migration assays. (**b**) Quantification of wound healing and transwell migration rate. ^*^P < 0.05 vs. 0 μg/mL of BPTS

Following treatment with the inhibitors for 12, 24 and 48 h, the migratory capacity of cells was observed and imaged. The migration distance of cells treated with the inhibitors was decreased compared with the control cells (Fig. 4). There was an inverse relationship between the concentration of the treatment used and the distance migrated. In the Transwell assay, the effect of BPTS on migration was examined. BPTS significantly decreased the number of invaded cells.

## Discussion

In modern medicine, surgery, chemotherapy and radiation are frequently used for treating patients with TNBC [25, 26]. However, these options are expensive, and patients exhibit high rates of recurrence and will frequently have a poor prognosis [27]. Therefore, an effective and economical treatment regimen for treating patients with TNBC is desirable. The study of using herbs and traditional Chinese medicines as alternatives or adjuvants to conventional treatments has gained traction, as a result of the possiblity of reduced side effects and improved therapeutic outcomes [28, 29]. BP is a traditional medicine that has been widely used in certain regions to prevent and treat many diseases, including, glioblastoma, prostate cancer, non-small cell lung cancer and colorectal cancer [30]. Treatment with Tu-bei-mu saponin preparations did result in some side effects in clinical studies at higher doses, including toxicity, allergic reactions and hemolysis. Previous works made a contribution to our work. Tubeimoside I, the respective major active ingredient of BPTS, may show antitumor activities [14]. Additonally, BPTS may initiate an allergic reaction when injected with Tu-bei-mu saponin [31].

In the present study, the effects and mechanisms of BPTS on MDA-MB-231 cells were examined. MDA-MB-231 cells are a well-studied TNBC cell line [32, 33]. MDA-MB-231 cells do not express ER, PR and Her2, and the majority of the frequently used therapeutics target one of these receptors. Therefore, this cell line is commonly used to research TNBC [34]. In the present study, the CCK-8 cell viability assay demonstrated that BPTS decreased the proliferation of MDA-MB-231 cells in a time-dependent and dose-dependent manner, with IC_50_ values varying from 5 to 15 μg/mL and also induced apoptosis. Furthermore, the migratory ability of MDA-MB-231 cells was decreased by BPTS treatment. The protein expression levels of p-PI3K, p-Akt and p-mTOR were all decreased following treatment with BPTS, and activation of the PI3K/Akt/mTOR signaling pathway has been demonstrated to promote proliferation, differentiation and survival of MDA-MB-231 cells [35].

The apoptotic pathways are the most important pathways resulting in cell death [36, 37]. However, resistence to apoptosis allows MDA-MB-231 cells to proiliefrate. In the present study, inhibiting the PI3K/Akt/mTOR signaling pathway induced apoptosis in agreement with previous studies [38, 39]. The expression of apoptosis markers, such as p53, caspases, Bax and Bcl-2 have all been demonstrated in MDA-MB-231 cells [40]. p53 is a downstream target of the PI3K/Akt/mTOR signaling pathway, and p53 upregulates Bax, which then activates caspases, resulting in apoptosis [41]. Inhibition of PI3K/Akt/mTOR pathway can affect multiple apoptosis inducing proteins [42]. Therefore it was hypothesized that the PI3K/Akt/mTOR signaling pathway was involved in BPTS-induced apoptosis.

To determine whether the PI3K/Akt/mTOR signaling pathway was able to affect migration in MDA-MB-231 cells, wound healing and Transwell migration assays were performed at a range of concentrations and treatment durations. Consistent with previous reports, inhibition of the PI3K/Akt/mTOR signaling pathway reduced the migration of cells. Migrating cells have been demonstrated to express increased levels of PI3K [43]. PI3K functions to recruit proteins containing Akt to the membrane, which can then be activated by PI3K-dependent kinase 1 and PI3K-dependent kinase 2 [44]. Activation of Akt in turn activates mTOR. Therefore, the PI3K/Akt/mTOR signaling pathway may function to regulate cell motility [45]. In MDA-MB-231 cells, the Akt family members mediate migration, proliferation, survival and protein synthesis [46]. In the present study, BPTS decreased Akt activation by decreasing the protein expression levels of PI3K, and this may have have modulated the migratory capacity of MDA-MB-231 cells.

Previous studies have demonstrated that several key molecules and signaling pathways regulate apoptosis in MDA-MB-231 cells [47, 48]. Among the signaling pathways, the PI3K/Akt/mTOR signaling pathway has been well-studied and has been demonstrated to regulate cell survival, proliferation and differentiation [41]. Inhibitors of the PI3K/Akt/mTOR signaling pathway may therefore prevent survival and induce apoptosis in TNBC cells. PI3K, was discovered > 20 years ago, and its role in metabolism, growth, survival and motility has been extensively studied [49]. Akt promotes cell survival and also abrogates the negative regulatory effect of the transcription factor NF-κB, resulting in an increase in transcription of anti-apoptotic and pro-survival genes [50]. mTOR is a highly conserved serine/threonine kinase, and its expression regulates metabolism and growth, and additionally integrates the signals from many stimuli [51]. Phosphorylation of these proteins is an important regulatory mechanism that results in conformational changes of many enzymes and receptors, resulting in their activation/deactivation [52]. In the present study, treatment of MDA-MB-231 cells with BPTS decreased the levels of phosphorylated PI3K, Akt and mTOR. These results suggest that BPTS may induce apoptosis of MDA-MB-231 cells through the PI3K/Akt/mTOR pathway.

BPTS could reduce migration and induce apoptosis of MDA-MB-231 cells through the PI3K/Akt/mTOR signaling pathway. Together, these data provide insight into the anti-tumor activity of BPTS and the underlying mechanisms. BPTS may be a potentially novel and effective treatment option for patients with TNBC.

## Conclusions

In conclusion, BPTS decreases proliferation, migration and apoptosis of MDA-MB-231 cells through the PI3K/Akt/mTOR pathway. These results provide a new theoretical basis for use of BPTS in a clinical setting, although additional in vivo studies are required. However, as only the MDA-MB-231 cell line was used, other TNBC cell lines need to be studied. Additional research into the mechanisms and potential clinical uses of BPTS are required.

## Data Availability

The datasets used and analyzed during the current study available from the corresponding author on reasonable request.

## Abbreviations

BP: Bolbostemma paniculatum (Maxim.) Franquet
BPTS: BP total saponins
DMSO: Dimethyl sulfoxide
Her2: Human epidermal growth factor receptor 2
LA: Luminal A
LB: Luminal B
TNBC: Triple-negative breast cancer
